# Highly efficient catalytic/sonocatalytic reduction of 4-nitrophenol and antibacterial activity through a bifunctional Ag/ZnO nanohybrid material prepared *via* a sodium alginate method†

**DOI:** 10.1039/c9na00075e

**Published:** 2019-07-02

**Authors:** Hicham Abou Oualid, Othmane Amadine, Younes Essamlali, Issam Meftah Kadmiri, Hicham El Arroussi, Mohamed Zahouily

**Affiliations:** VARENA Center, MAScIR Foundation Rabat Design, Rue Mohamed El Jazouli, Madinat El Irfane 10100-Rabat Morocco m.zahouily@mascir.com +212661416359; Laboratoire de Matériaux, Catalyse et Valorisation des Ressources Naturelles, URAC 24, FST, Université Hassan II-Casablanca BP, 146 20650 Morocco; Green Biotechnology Center, Moroccan Foundation for Advanced Science, Innovation and Research, Rabat Design Center Rabat Morocco

## Abstract

In this work, a bifunctional nanohybrid silver/zinc oxide material (Ag/ZnO) has been synthesized by a rapid route using sodium alginate simultaneously as a sacrificial template and silver reducing agent. The obtained samples were characterized by X-ray diffraction (XRD) and Fourier transform infrared (FTIR) spectroscopy, scanning electron microscopy (SEM), transmission electron microscopy (TEM), solid diffuse reflectance and liquid state UV-visible spectroscopy (DRS, UV-visible), and nitrogen adsorption–desorption analysis (BET–BJH). The XRD patterns showed that the Ag/ZnO sample is composed of a hexagonal zinc oxide structure with cubic metallic silver (Ag°). SEM micrographs exhibited a porous structure which was confirmed by BET–BJH methods to be mesoporous. The Ag/ZnO material was used as a nanocatalyst in the conversion of 4-nitrophenol (4-NP) to 4-aminophenol (4-AP) as well as an antibacterial agent against *Escherichia coli* and *Staphylococcus aureus*. It was found that an efficient 4-NP reduction to 4-AP in the presence of NaBH_4_ shows a rate constant of 0.418 min^−1^ under ultrasonic energy and 0.316 min^−1^ without ultrasonic energy. Both the catalysis reaction and antibacterial activity analysis were conducted in water solution and showed a synergetic effect of metallic silver loading.

## Abstract 2

In this work, a bifunctional nanohybrid silver/zinc oxide material (Ag/ZnO) has been synthesized by a rapid route using sodium alginate simultaneously as a sacrificial template and silver reducing agent.

## Graphical abstract

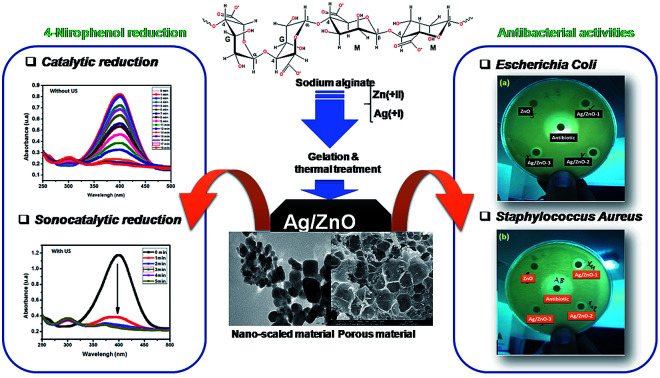

## 1. Introduction

Zinc oxide is considered as a natural engineering material^1–5^ used in several applications owing to its catalytic,^2^ electrical,^3^ optoelectronic^4^ and photochemical properties.^5^ ZnO is a semiconductor material with a direct wide bandgap (3.37 eV) and a large exciton binding energy (60 meV),^6^ and can be prepared by numerous methods such as hydrothermal,^7,8^ sol gel,^9–11^ surfactant free in-air and microwave methods.^8^ The main disadvantages of using these methods are the harsh synthesis conditions, the high temperature of treatment and the high cost of the starting compounds. Recently, the gelation method using natural polysaccharides such as alginate,^12^ chitosan,^13^ starch^14,15^ and cellulose^16^ for the preparation of nanoparticles has been introduced for the replacement of conventional methods. The large surface area, structural morphology, nontoxicity, low price, and ease of processing are the main advantages of this method of synthesis. Since the gelation method using polysaccharides is simple, fast, and economical and does not require special instruments, it offers the best choice for large scale green and economical synthesis of nanoparticles. However, ZnO has been widely used as a catalyst or catalyst support of several metal nanoparticles for various organic transformations.^6,17–20^ Moreover, it was reported that impregnation of ZnO by transition metals increases its catalytic activity and extends the light absorption spectrum of ZnO into the visible region. Among transition metals, Ag nanoparticles have attracted great interest, because they significantly improve the ZnO photocatalytic performance. Thus, Zhai *et al.* demonstrated the effect of the ZnO photocatalytic activity in the presence of silver for the degradation of rhodamine B under solar irradiation.^21^ In another study, Liang *et al.* conducted a comparative study by doping Ag on ZnO and TiO_2_ in order to degrade rhodamine B under UV irradiation.^22^ Bouzid *et al.* also reported the degradation of methylene blue in the presence of ZnO doped with different percentages of silver. This study led Bouzid *et al.* to conclude that the best degradation under UV irradiation took place when doping ZnO with 1% of Ag. On the other hand, Ag/ZnO has attracted considerable interest in recent times because of its promising biological activity as an antibacterial agent.^23–25^ In fact, Ag/ZnO has been used as an antibacterial material against *E. coli* and *S. aureus*.^26^ The structural morphology and silver loading of the nanocomposite affect the antibacterial performance.^27,28^

In recent times, 4-nitrophenol has been one of the most dangerous pollutants usually present in industrial effluents.^29^ According to the United States Environmental Protection Agency, 4-NP is one of the most toxic and most hazardous pollutants.^30^ Therefore, the reduction of *p*-nitrophenol to *p*-aminophenol is of great importance, and the obtained product can be used as an intermediate in the synthesis of several pharmaceutical compounds such as paracetamol and acetanilide.^31^ The hydrogenation of 4-NP has been widely investigated using high temperature and high hydrogen pressure.^22,32^ However, these reactions generally suffer from high reaction temperature, long reaction time and also the corrosion of the reaction vessels, which limit their further applications. To overcome these drawbacks, another catalytic route has been developed for the reduction of 4-NP to 4-AP using sodium borohydride in aqueous medium under mild conditions. During this reduction, various catalysts, such as metal oxides (*e.g.*, ZnO,^33^ SiO_2_,^34^ and TiO_2_ (ref. 33) *etc.*), metallic nanoparticles (*e.g.*, Ag,^35^ Pd,^36^ Pt,^37^*etc.*) and hybrid metal oxide/metal materials (Ag/TiO_2_,^38^ Au/TiO_2_,^39,40^ Au/ZnO,^41^ Au/Fe_2_O_3_,^42^*etc.*), have been used to speed up the reduction process. Therefore, the design of suitable heterogeneous catalysts for reduction of *para*-NP to *p*-AP has been an important area of research. Herein, we report the synthesis of nanohybrid Ag/ZnO *via* a green and rapid route by using sodium alginate as a cross-linker and reducing agent. The structural and morphological properties of the Ag/ZnO nanohybrid were fully characterized by XRD, SEM, TEM and N_2_ adsorption/desorption. The catalytic activity of the Ag/ZnO nanohybrid was evaluated by the reduction of 4-nitrophenol to 4-aminophenol. And the antibacterial activity of the Ag/ZnO nanohybrid was also evaluated. To the best of our knowledge, there are no papers that have examined the catalytic activity for reduction of 4-nitrophenol and the antibacterial acitivity against *E. coli* and *S. aureus* using Ag/ZnO prepared by the gelation method.

## 2. Experimental section

### 2.1. Chemicals and medium

Zinc nitrate hexahydrate (Zn(NO_3_)_2_ 6H_2_O, 98%), silver nitrate (AgNO_3_, 99%), 4-nitrophenol and sodium borohydride (NaBH_4_) were purchased from Aldrich and used as precursors. Sodium alginate was purchased from Aldrich and used as supplied. Deionized water was used in all experiments. *Escherichia coli* (ATCC-8739) and *Staphylococcus aureus* (ATCC-6538) were used for the bacterial studies.

### 2.2. Preparation of Ag/ZnO nanohybrid materials

**Fig. 1:**
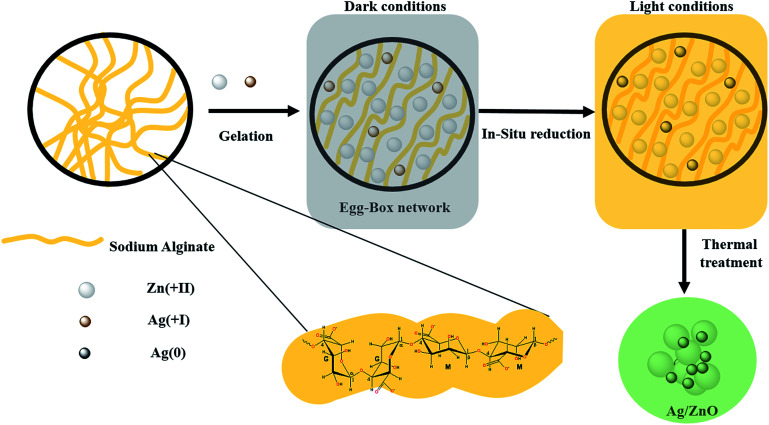
Schematic representation of the chemical route used for the synthesis of Ag_*x*_/ZnO.

The preparation of all materials follows the same procedure as that described in our previous paper.^43^ Typically, 1 g of sodium alginate powder was dissolved in 100 mL of deionized water at room temperature for 2 hours, resulting in a transparent and viscous gel solution. 2.97 g of hexahydrate zinc nitrate and an appropriate amount of silver nitrate were dissolved together in 100 mL of deionized water to obtain different percentages of silver (1, 2 and 3 wt%). Then a viscous solution of alginate was added dropwise to the above solution over 2 hours under dark conditions. The reaction proceeded overnight under slow and constant stirring to avoid a deterioration of beads. The beads were immediately formed in the dark and then changed to black-brown after the removal of the diaphragm. Then, the beads were separated from the solution and washed several times with distilled water to remove the excess sodium and nitrate ions on their surfaces. Afterward, the beads were dried at room temperature for 24 h and then thermally treated at 500 °C for 10 hours (the adequate calcination temperature was defined using TGA analysis (Fig. A1 ESI†)). The as-prepared material has the aspect of a fine and gray powder (Fig. A2 ESI†). The above materials were labeled as Ag_*x*_/ZnO, where *x* represents the content of Ag (wt%) in the materials. Fig. 1 schematizes the described synthesis procedure.

### 2.3. Characterization

X-ray diffraction patterns were obtained at room temperature on a Bruker AXS D-8 diffractometer using Cu-Kα radiation in Bragg–Brentano geometry (*θ*–2*θ*). Fourier transform infrared (FT-IR) spectra of the samples in KBr pellets were measured on a Bruker Vector 22 spectrometer. Thermogravimetric analysis (TGA) was conducted in air using a TA Instruments Q500 apparatus, with a 10°C min^−1^ heating rate from 25 to 1000 °C. Scanning electron microscopy (SEM) images were recorded on an FEI Quanta 200 microscope after carbon metallization. TEM micrographs were obtained on a Tecnai G2 microscope at 120 kV. The gas adsorption/desorption data were collected using a Micromeritics 3Flex surface characterization analyzer using N_2_. Prior to N_2_ sorption, all samples were degassed overnight at 150 °C. The specific surface areas were determined from the nitrogen adsorption/desorption isotherms (at −196 °C), using the BET (Brunauer–Emmett–Teller) method. Pore size distributions were calculated from the N_2_ adsorption isotherms with the ‘‘classic theory model’’ of Barrett, Joyner and Halenda (BJH). The catalytic tests were conducted in aqueous solution using a JENWAY-Model 6700 spectrophotometer. Elemental analyses were realized using inductively coupled plasma atomic emission spectrometry (ICP AES; Ultima 2 – Jobin Yvon).

### 2.4. 4-Nitrophenol reduction procedure

The 4-nitrophenol reduction was carried out in a beaker (100 ml). In a standard reaction, 0.17 mg of 4-NP and 47.28 mg of NaBH_4_ were added to 50 ml of deionized water. Then, the solution was taken out at given time intervals and filtered to remove the catalyst particles completely. The analysis of the concentration of 4-NP in the filtered solution was carried out using a UV spectrophotometer. The characteristic maximum absorbance of 4-NP at 401 nm was used as an indicator of the remaining 4-NP, and the absorbance at 300 nm was utilized to identify the 4-AP.

### 2.5. Antibacterial test study

The antibacterial activity of the prepared samples was studied using a disc diffusion method with two strains of the selected bacteria – *Staphylococcus aureus* (*S. aureus*) and *Escherichia coli* (*E. coli*) – and the zone of inhibition was analyzed. Before inoculation, the pathogenic bacteria were cultured individually in Muller-Hinton broth at 37 °C for 24 h. A solid culture was prepared by mixing 2 g of beef extract, 3.5 g of casein acid hydrolyte, and 3 g of starch in 200 mL of distilled water. A total of 20 mL of microbial culture was uniformly distributed on the plate. Sterile paper discs saturated with a solution of 10 μL of sample (ZnO, Ag_0.84_/ZnO, Ag_1.68_/ZnO, or Ag_2.98_/ZnO) were gently placed over the test organism seeded plates. An amikacin 30 mg antibiotics disc and a disc loaded with 100 mL of HCl (4 N) were used as positive and negative controls, respectively. The plates were incubated for 24 h at 37 °C in a bacteriological incubator. The zone of inhibition was then measured and recorded. Each experiment was made in duplicate, and the inhibition zone was measured with a vernier caliper. Then, the study of the cell reduction activity of the prepared samples was performed using the colony count method. With 200 μL of sample, an approximate number of 108 CFU ml^−1^ of the *S. aureus* and *E. coli* bacteria in Muller-Hinton broth were added and incubated at 37 °C on a shaking platform at 250 rpm for 24 h. A control broth without the samples was also tested. The number of bacterial colonies (CFU) was counted and was interpreted in terms of reduction percentage.

## 3. Results and discussion

### 3.1. Characterization of the Ag/ZnO nanohybrid material

**Fig. 2:**
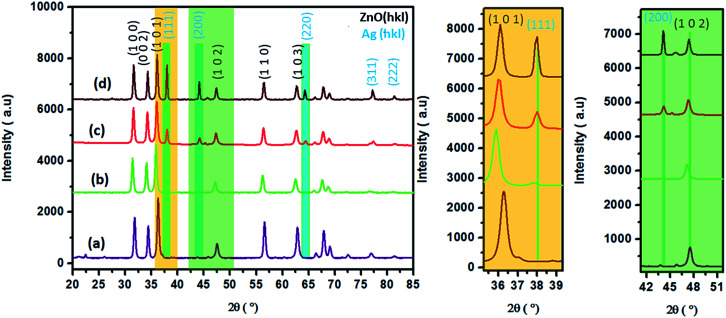
XRD patterns of pure ZnO (a), Ag_0.84_/ZnO (b), Ag_1.68_/ZnO (c) and Ag_2.98_/ZnO (d) nanohybrid materials (left). A zoomed-in view of the (101) and (102) peaks (right).

The phase purity and composition of the products attained by the sodium alginate method were examined by XRD. Fig. 2 displays the powder XRD patterns of the samples (Ag_0.84_/ZnO, Ag_1.68_/ZnO and Ag_2.98_/ZnO nanomaterials). The recorded diffraction peaks can be categorized into two sets. Peaks without blue highlighting have good matches with Bragg reflections of the standard wurtzite structure with space group *P*6_3_*mc* (186) (*a* = 0.3249 nm, *c* = 0.5206 nm) of ZnO (JCPDS file no. 00-36-1451), while the other ‘blue-highlighted’ peaks agree well with the face-centered cubic (FCC) lattice with space group *Fm*3̄*m* (225) (*a* = 0.4087 nm) of metallic silver (JCPDS file no. 01-080-4432). In the case of pure ZnO, the major peaks are detected at 2*θ* values of 31.82, 34.47, 36.29, 47.56, 56.53, 62.89, 66.35, 67.96, 69.15, 72.56 and 77.46°, which correspond to the (100), (002), (101), (102), (110), (103), (200), (112), (201), and (202) crystalline planes of the hexagonal ZnO structure.^44,45^ Moreover, no crystalline phase ascribed to silver species was observed in Ag_0.84_/ZnO, probably due to the small amount of silver. However, the diffractograms of Ag_1.68_/ZnO and Ag_2.98_/ZnO samples showed the presence of a secondary phase in which the diffraction peaks correspond to metallic silver. The average crystallite sizes of all samples estimated according to the Debye–Scherrer equation (eqn (1)) are given in Table 1.1
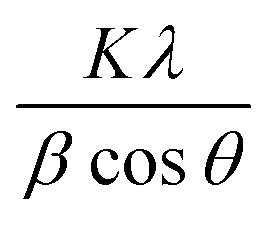
where *K* and *λ* are the Scherrer shape factor (taken as 0.89) and the X-ray wavelength of CuK_α_ radiation (taken as 1.54 Å), respectively, *θ* is the Bragg angle, and *β* is the pure line broadening.

**Table 1:** Lattice parameter and crystallite size of the nanohybrid samples

Samples	Lattice parameter (Å)	Volume cell	Crystallite size^a^/nm	Reference
ZnO	3.242	47.30	19.7	48
	5.196			
Ag_0.84_/ZnO	3.250	47.65	20.5	This work
	5.200			
	4.087	68.28	49.3	
Ag_1.68_/ZnO	3.248	47.61	21.0	
	5.207			
	4.086	68.21	50.2	
Ag_2.98_/ZnO	3.248	47.60	22.1	
	5.208			
	4.085	68.20	53.7	

a Calculated from the Scherrer equation.

An increase in the crystallite size is observed from 19.7 nm for ZnO, to 20.5, 21.0 and 22.1 nm for Ag_0.84_/ZnO, Ag_1.68_/ZnO and Ag_2.98_/ZnO, respectively. This size augmentation is probably due to the presence of metallic silver. It's worth noting that the XRD pattern of the three samples displayed a slight shift of ZnO peaks, which indicated lattice expansion due to the presence of metallic silver.

**Fig. 3:**
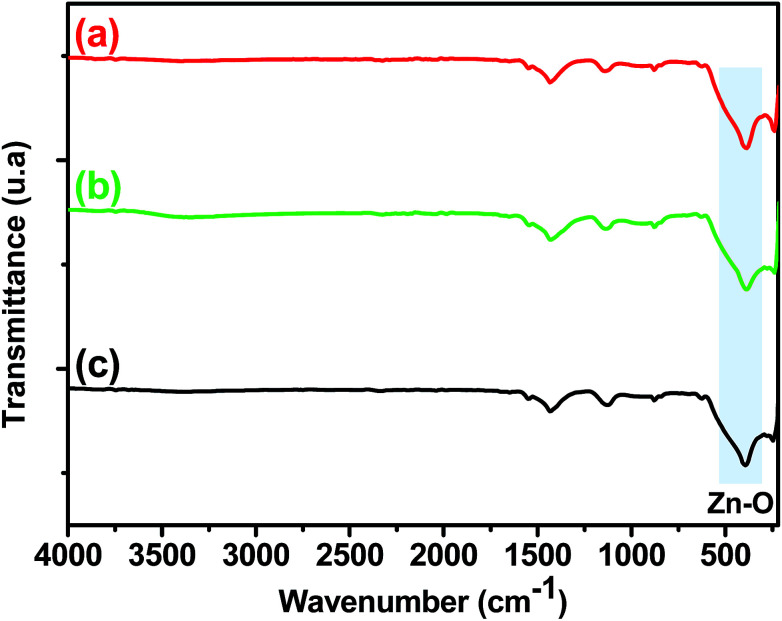
FT-IR spectra of (a) Ag_0.84_/ZnO, (b) Ag_1.68_/ZnO and (c) Ag_2.98_/ZnO in the range of 400–4000 cm^−1^.

The nanohybrid materials obtained were characterized by FT-IR spectroscopy in order to determine the characteristic bands of all the samples (Fig. 3). In this figure, the appearance of a characteristic vibration-sensitive band at about 401 cm^−1^ characteristic of Zn–O is observed.^46–48^ Furthermore, the three samples exhibit a similar shape with the presence of the fingerprint of zinc oxide, which demonstrates the conservation of the base framework of the ZnO structure after silver loading. Meanwhile, the band characteristic of the hydroxyl group at about 3290 cm^−1^ disappears, maybe due to the elimination of the hydroxyl groups from sodium alginate after its decomposition.^49,50^ Moreover, the Zn–O band vibrations in the case of Ag/ZnO and in the case of pure ZnO elaborated in a previous study^43^ were compared. As shown in Fig. A3 ESI,† pure ZnO and Ag/ZnO exhibit a stretching vibration mode at about 392 and 401 cm^−1^, respectively. This shift is undoubtedly due to the surface interaction between ZnO particles and metallic silver.^51^

**Fig. 4:**
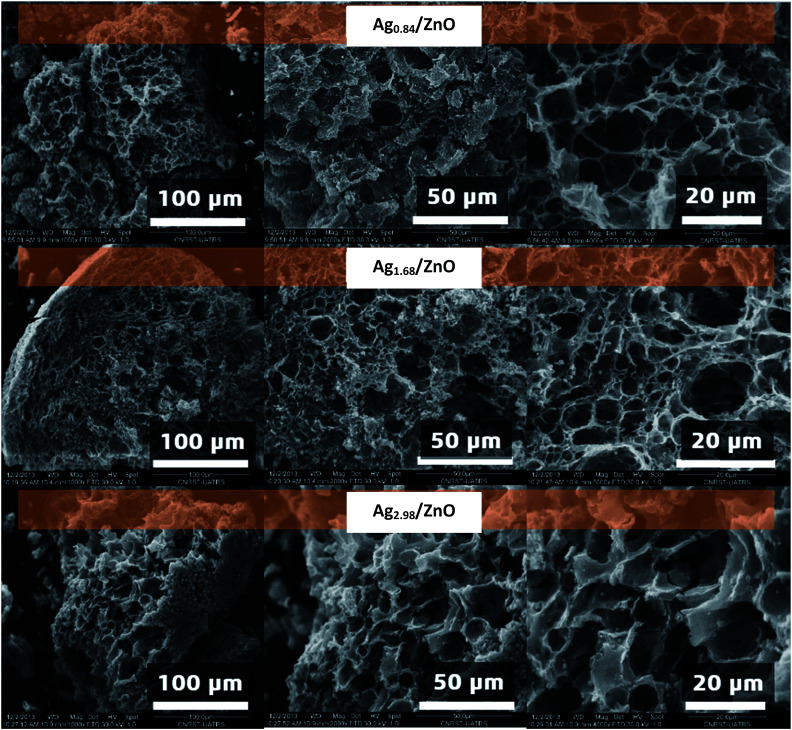
SEM images under different magnifications of Ag_0.84_/ZnO, Ag_1.68_/ZnO and Ag_2.98_/ZnO nanohybrid materials.

In order to better elucidate the morphology of the nanohybrid materials obtained after thermal treatment at 500 °C, scanning electron microscope analysis of Ag_0.84_/ZnO, Ag_1.68_/ZnO and Ag_2.98_/ZnO was carried out (Fig. 4). The micrographs show that all analyzed samples exhibit a porous structure characterized by the presence of a network of interconnected pores of micrometric sizes. It should be noted that the porous structure of the as-prepared materials is mainly due to the outflow of the gases generated during the oxidative degradation of the organic material during the thermal treatment process.^52,53^ Also, we noticed that the microstructure of all materials was affected by the presence of metallic silver and alginate decomposition, as explained in our previous work.^43^ As the amount of silver was increased, the decomposition became less fast.^54^ In fact, the silver cation affects both carboxylic and hydroxyl groups. Hence in the case of the zinc cation, the complexation is focused only at the carboxyl group.

**Fig. 5:**
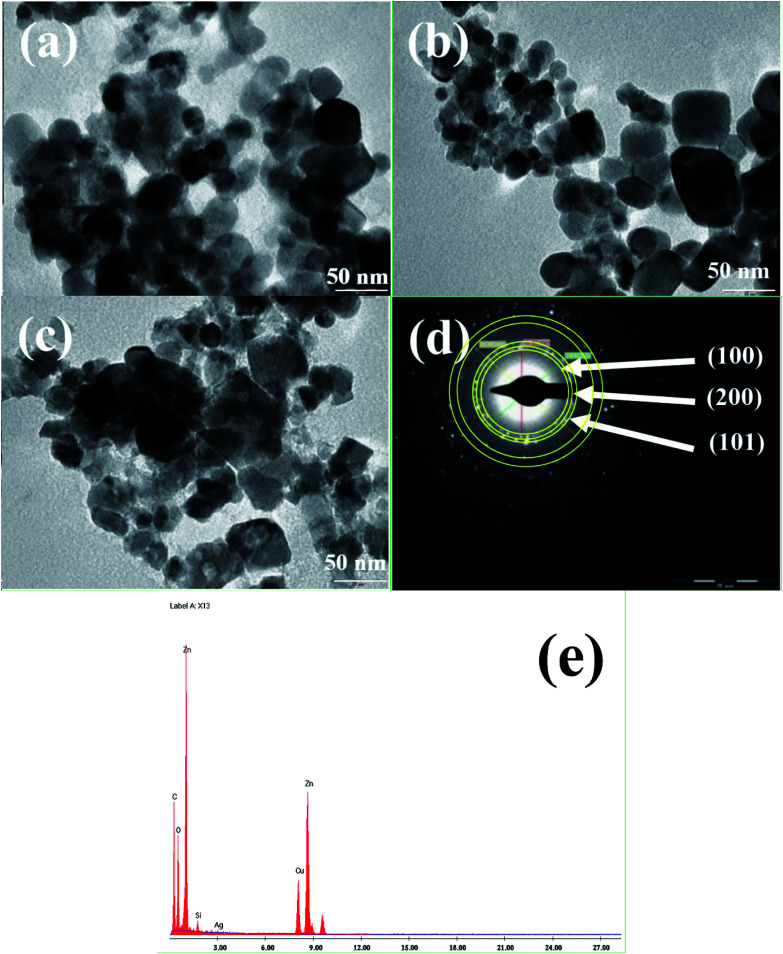
TEM images and EDAX analysis of Ag_0.84_/ZnO (a), Ag_1.68_/ZnO (b) and Ag_2.98_/ZnO (c) respectively; electron diffraction analysis (d) and EDAX analysis (e) of Ag_2.98_/ZnO.

To facilitate an in-depth investigation of the textural properties of all samples, a nitrogen adsorption–desorption analysis at the temperature of liquid nitrogen (77 K) was investigated. The adsorption–desorption isotherms of materials Ag_0.84_/ZnO, Ag_1.68_/ZnO and Ag_2.98_/ZnO are shown in Fig. 5. According to this figure, the isotherms of all materials have a similar appearance and show the existence of micropores and mesopores.^59^ All these isotherms correspond to type II according to the Brunauer–Deming–Deming–Teller (BDDT) classification system,^7^ which characterizes the formation of a mesoporous material (Fig. A4 ESI†).

The textural parameters of the prepared materials are summarized in Table 2. The specific surface areas of Ag_0.84_/ZnO, Ag_1.68_/ZnO and Ag_2.98_/ZnO are of the order of 7.87, 9.12, and 8.78 m^2^ g^−1^, respectively. These results show that the silver loading on ZnO materials does not affect significantly their specific surface areas. The pore size distributions of Ag_0.84_/ZnO, Ag_1.68_/ZnO and Ag_2.98_/ZnO materials determined by the BJH method are shown in Fig. 5b, d and f, respectively. The materials have a homogeneous distribution with average values of around 2.9, 3.12 and 3.22 nm for Ag_0.84_/ZnO, Ag_1.68_/ZnO and Ag_2.98_/ZnO, respectively. From these results, it can be concluded that Ag_0.84_/ZnO, Ag_1.68_/ZnO and Ag_2.98_/ZnO correspond to mesoporous materials (between 2 and 50 nm). Pore volumes were found to be 0.0083, 0.0092 and 0.0087 cm^3^ g^−1^ for Ag_0.84_/ZnO, Ag_1.68_/ZnO and Ag_2.98_/ZnO, respectively.

**Table 2:** Textural properties of the Ag/ZnO nanohybrid materials

Samples	Surface area (m^2^ g^−1^)	BJH pore volume (cm^3^ g^−1^)	BJH pore size distribution (nm)
Ag_0.84_/ZnO	7.8759 ± 0.2277	0.0083	2.92
Ag_1.68_/ZnO	9.1207 ± 0.2227	0.0092	3.12
Ag_2.98_/ZnO	8.7858 ± 0.2244	0.0087	3.22

**Fig. 6:**
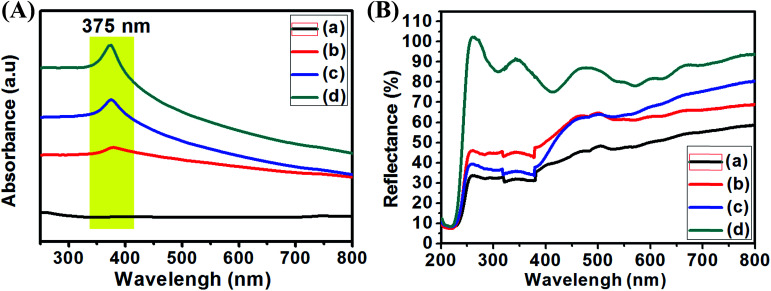
UV-visible absorption of liquid samples (A) and solid diffuse reflectance (B) of pure ZnO (a), Ag_0.84_/ZnO (b), Ag_1.68_/ZnO (c) and Ag_2.98_/ZnO (d).

Transmission electron microscopy (TEM) was conducted to investigate the shape and the size of all the prepared nanohybrid materials. The TEM images revealed that Ag_0.84_/ZnO (Fig. 5a), Ag_1.68_/ZnO (Fig. 6b) and Ag_2.98_/ZnO (Fig. 6c) are well nanostructured with a quasi-spherical morphology. It was found that the average sizes of the nanoparticles were about 25, 26 and 24 nm for Ag_0.84_/ZnO, Ag_1.68_/ZnO and Ag_2.98_/ZnO, respectively. Relating to the particle sizes calculated earlier using the Debye–Scherrer equation (eqn (1)), where the average crystallite size was found to be 20, 21 and 22 nm for Ag_0.84_/ZnO, Ag_1.68_/ZnO and Ag_2.98_/ZnO, respectively, the particles sizes have the same tendency because the slight difference of average sizes between crystallites and nanoparticles is normal since it is well established that crystallites are always smaller than or equal to nanoparticles in size.^55^ Selected area electron diffraction (SAED) was also used to investigate the local structure of Ag/ZnO. Fig. 5d shows the SAED pattern of Ag_2.98_/ZnO. This image shows that the ZnO nanocrystals are less crystalline and possess a hexagonal wurtzite structure, in which the main diffraction rings are perfectly indexed to the same positions as those from hexagonal ZnO (space group *P*6_3_*mc*, JCPDS card no. 36-1451). Nevertheless, there is no clear ring characteristic to cubic silver due possibly to the SAED resolution and/or small silver amount. On the other hand, EDS analysis of Ag_2.98_/ZnO (Fig. 5e) was conducted to confirm the existence of silver in the prepared material. Thereafter, ICP analysis was performed to determine the mol% of silver incorporated in the final materials. From this analysis, we found that the silver concentrations are 0.84, 1.68 and 2.98 mol% for Ag_0.84_/ZnO, Ag_1.68_/ZnO and Ag_2.98_/ZnO, respectively.

**Fig. 7:**
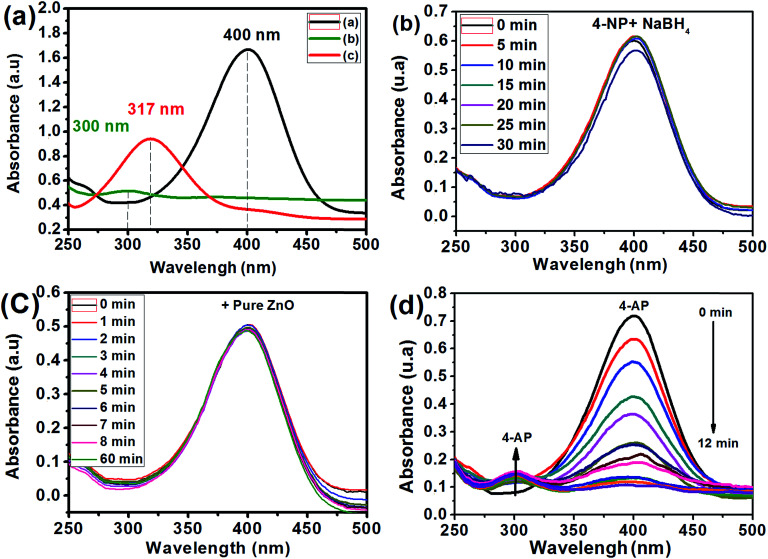
UV-vis spectra of aqueous solutions of 4-NP (a) with and without NaBH_4_, (b) UV-vis spectral evolution of solutions of 4-NP in the presence of NaBH_4_ without any catalyst, (c) in the presence of pure ZnO and (d) in the presence of the Ag_2.98_/ZnO catalyst.

The UV-vis absorption spectra of pure ZnO and Ag/ZnO nanohybrid materials were recorded at room temperature in the liquid and solid state in order to examine the optical properties and silver influence on the as-prepared materials. The obtained results are shown in Fig. 6A and B. Fig. 7a shows that the absorption peak characteristic of Ag/ZnO was observed at 375 nm from the surface plasmon resonance (SPR) absorption of Ag nanoparticles.^56^ In addition, the absorbance peak increases in the higher wavelength region with increasing silver amount in ZnO. According to Fig. 6B, the values of diffuse reflectance of the as-prepared nanohybrids increase with the increase of silver loading, especially in the far and near UV (200–400 nm) and UV-visible (400-800) regions.^57^

### 3.2. Catalytic and sonocatalytic activity

The Ag/ZnO samples were evaluated as nanocatalysts for 4-NP reduction in the presence of NaBH_4_. Fig. 7 shows the absorbance peak of the 4-NP.

**Fig. 8:**
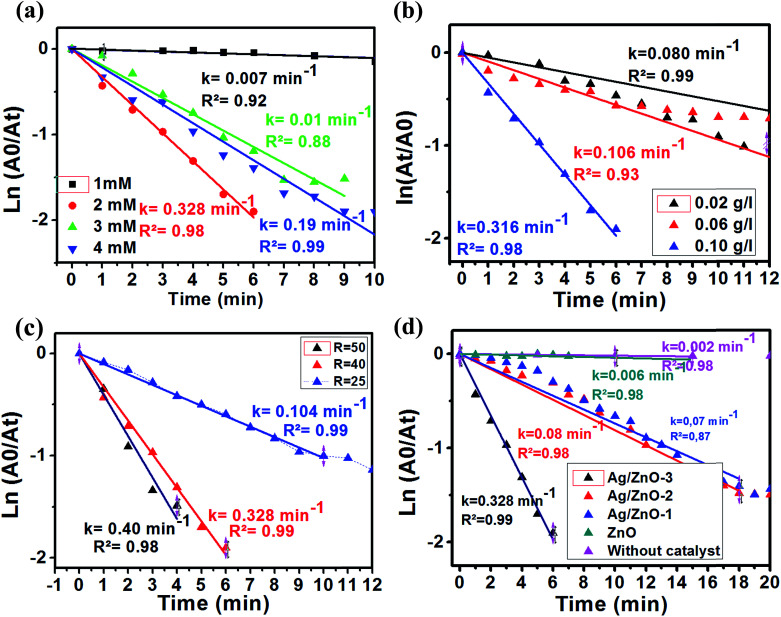
Logarithm of the absorbance at 400 nm *versus* reduction time of 4-NP under different conditions: 4-NP concentration (a), [NaBH_4_/4-NP] molar ratio (b), catalyst amount (c) and material types (d).

We studied the influence of 4-NP concentration on the reduction of 4-NP in the presence of Ag_2.98_/ZnO as a catalyst (0.1 mg L^−1^) and molar ratio (NaBH_4_/4-NP) = 40 at room temperature. Firstly, concentrations of 1, 2, 3 and 4 mM were employed as initial concentrations of 4-NP. Fig. A6 ESI† shows the evolution of the absorption spectrum at a maximum wavelength of 400 nm, which corresponds to the absorption of 4-nitrophenolate. The ln(*A*_0_/*A*_t_) plot as a function of time for the four previously mentioned concentrations is shown in Fig. 8a. As seen in this figure, it was found that the rate constant is higher in the case of 2 mM (0.328 min^−1^) and very low at 1 mM (0.007) concentration of 4-NP. From these results, the ideal concentration of 4-NP for future experiments is 2 mM.

**Fig. 9:**
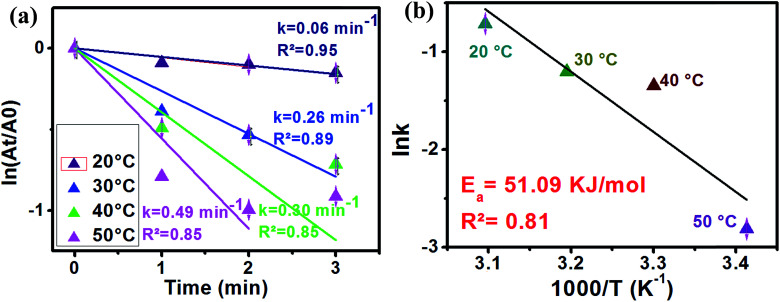
The logarithm of the absorbance at 400 nm *vs.* reduction time of 4-NP at different temperatures (a) and ln *k vs.* 1000/*T* plot (b).

The influence of the mass concentration of the catalyst was studied. The reaction was conducted at different mass concentrations of Ag_2.98_/ZnO ranging from 0.02 to 0.1 g L^−1^ at room temperature. The amount of NaBH_4_ was set in such a way as to have a molar ratio [NaBH_4_/4-NP] of 40. We should note that the reaction was started instantaneously after the addition of the Ag_2.98_/ZnO catalyst, which quickly results in a change of the yellow color, indicating the disappearance of the 4-NP. The solutions become transparent after a few minutes. For each concentration of Ag_2.98_/ZnO, we plotted the Abs(*t*) = *f*(*t*) curves in order, to follow the evolution of the absorbance of 4-nitrophenolate as a function of time (Fig. A7 ESI†). Fig. 9b shows the ln(*A*_t_/*A*_0_) plot as a function of time and mass concentrations of Ag_2.98_/ZnO (0.02, 0.08 and 0.1 g L^−1^). According to this figure, that rate constant of 4-NP to 4-AP increases from 0.08 to 0.315 min^−1^ with the increase of the catalyst mass from 0.02 to 0.1 g L^−1^.

Considering the great importance placed by several studies in the literature on the influence of the [NaBH_4_/4-NP] molar ratio on the catalytic efficiency of the reduction of 4-NP into 4-AP, a study was carried out by testing the Ag_2.98_/ZnO nanohybrid at different [NaBH_4_/4-NP] molar ratios (Fig. A8 ESI†). Fig. 9c shows the ln(*A*_0_/*A*_t_) plot as a function of experiment time at molar ratios of 25, 40, and 50, with a 4-NP concentration of 2 mM and a catalyst mass concentration of 0.1 g L^−1^ at room temperature. The plot of the results shows that the increase of the [NaBH_4_/4-NP] molar ratio from 25 to 50 induces an increase in the rate constant from 0.104 to 0.4 min^−1^, respectively. This result can be explained by the presence of an excess of the reducing agent, which promotes the diffusion of BH_4_^−^ ions on the catalyst surface by accelerating the reduction of the diffused 4-NP.^42,58^ Thus, the influence of the [NaBH_4_/4-NP] molar ratio is determinant. Since the molar ratio of [NaBH_4_/4-NP] = 50 is uncontrollable and ends in three minutes, a molar ratio of [NaBH_4_/4-NP] = 40 was chosen for the subsequent tests.

The influence of the percentage of silver in the Ag/ZnO nanohybrid on the catalytic efficiency of the 4-NP reduction reaction into 4-AP was studied as a function of time. For an overall comparison, all the catalytic tests under the same conditions (initial concentration: 2 mM, ratio [NaBH_4_/4-NP] = 40) are presented. Fig. 8d shows the ln(*A*_0_/*A*_t_) plot as a function of time, in the absence of any catalyst, and in the presence of pure ZnO, Ag_0.84_/ZnO, Ag_1.68_/ZnO and Ag_2.98_/ZnO. According to Fig. 8d, the 4-NP reduction reaction into 4-AP is very slow without catalyst addition (*k* = 0.002 min^−1^); this result is confirmed in several studies.^29,38,59^ However, when pure zinc oxide was prepared according to our previous work,^60^ the reaction rate wasn't significantly affected (*k* = 0.006 min^−1^). It is thus deduced that, under these conditions, pure zinc oxide does not effectively catalyze the 4-NP reduction reaction under these conditions. The same results were obtained by Chanu *et al.*,^61^ who showed that the catalytic activity only manifests itself in the presence of a coupling agent of gold and zinc oxide. For this reason, the three nanohybrids, Ag_0.84_/ZnO, Ag_1.68_/ZnO and Ag_2.98_/ZnO, were tested. From the plot of ln(*A*_0_/*A*_t_) as a function of time (Fig. 8a), it is clearly seen that silver has an interesting effect on the catalytic efficiency for the 4-NP reduction. In this case, the rate constant increases from 0.006 min^−1^ in the presence of ZnO to 0.07, 0.08 and 0.328 min^−1^ in the presence of Ag_0.84_/ZnO, Ag_1.68_/ZnO and Ag_2.98_/ZnO, respectively. These results are more promising than those obtained for the Ag@TiO_2_ composite catalyst (% Ag = 9.2) characterized by a rate constant of 0.088 min^−1^, as well as those for the ZnO–SiO_2_–Ag nanohybrids where the rate constant is 0.16 min^−1^. This catalytic activity of the Ag/ZnO is due to the nature of the semiconductor used and the nature of the metal as well as the charge transfer in the semiconductor/metal interface of the composite.^38,42,62,63^ It was recently found that 3D Ag/ZnO assemblies synthesized by other methods were used for the removal of 4-nitrophenol by photodegradation.^63^ Another Ag/ZnO composite was also fabricated by using polyacrylamide-gel methods, and the catalyst exhibits a good photocatalytic performance.^64^ Lungu *et al.* reported the preparation of Ag/ZnO prepared with chemical and mechanical deposition and investigated its antimicrobial activity. From the results of this study, the authors showed that the Ag/ZnO prepared by the chemical method in the presence of a capping agent (carboxymethyl cellulose) gives small size Ag/ZnO nanoparticles and prevents the occurrence of nanoparticle agglomeration, which increases the antimicrobial activity toward a broad range of bacterial and fungal strains due to the high surface to volume ratio of nanoparticles.^65^

In order to estimate the activation energy of our sample, the effect of the reaction temperature variation was investigated. In this study, Ag_2.98_/ZnO was selected for this part of the study. Fig. 9a shows the plot of ln(*A*_t_/*A*_0_) as a function of time according to the first-order model of the reaction temperatures ranging from 20 to 50 °C.

As shown in Fig. 9a, an increase in the temperature from 20 to 50 °C is observed, accompanied by an increase in the reaction rate of 4-nitrophenol reduction to 4-aminophenol. In fact, the rate constant was 0.06, 0.026, 0.03 and 0.49 min^−1^ for 20, 30, 40 and 50 °C, respectively. On the other hand, we calculated the activation energy using an Arrhenius diagram plot. Fig. 9b shows the Arrhenius plot of the rate constants of 4-nitrophenol reduction to 4-aminophenol with an excess of NaBH_4_ in the presence of Ag_2.98_/ZnO. The activation energy (*E*_a_) was evaluated by plotting ln(*k*) as a function of 1000/*T* according to the Arrhenius equation (eqn (2)).2
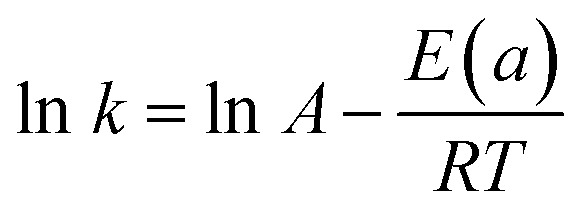
where *k* is the velocity constant, *R* is the real gas constant (8.314 J mol^−1^ K) and *A* is a constant. *E*(*a*) corresponds to the slope of the plotted line.

**Fig. 10:**
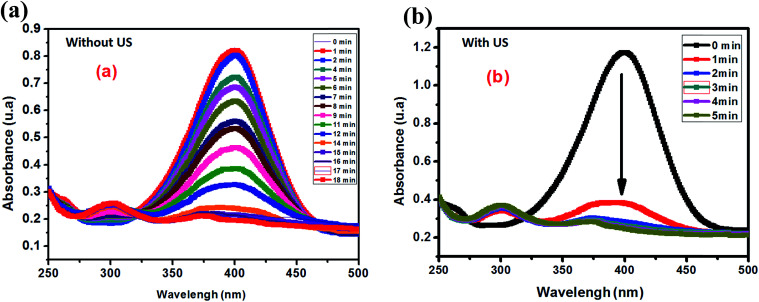
Evolution of the UV-visible spectrum of the reduction of 4-NP: (a) without ultrasound and (b) with ultrasound activation.

**Fig. 11:**
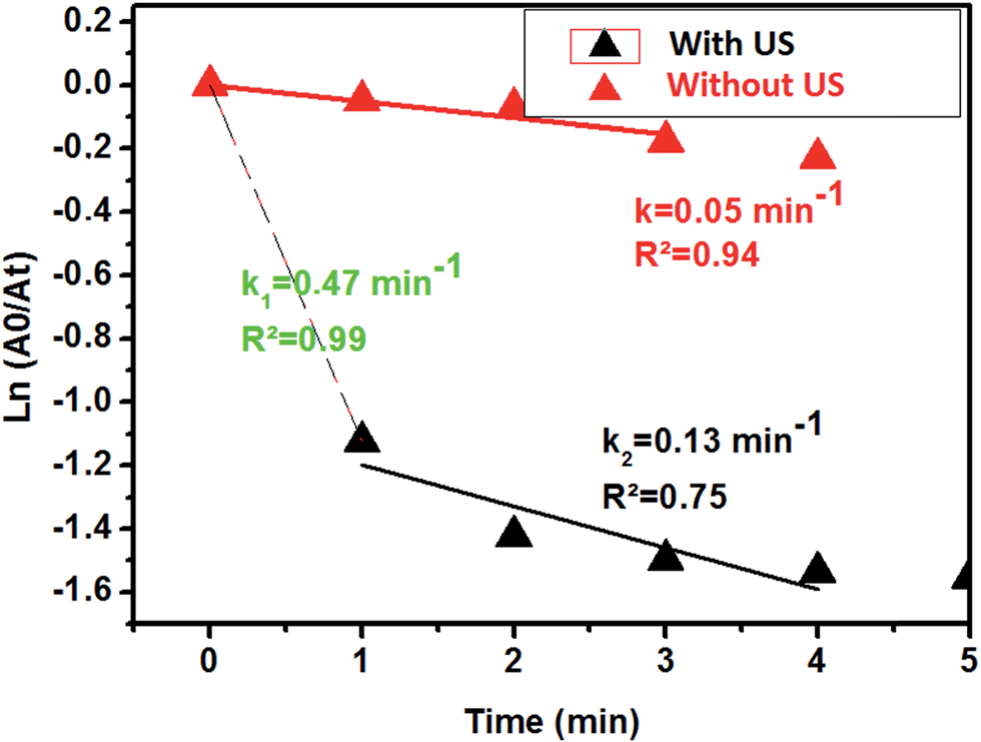
Evolution of the UV-visible spectrum of the reduction of 4-NP with ultrasound and without ultrasound in the presence of the Ag_2.98_/ZnO catalyst.

**Fig. 12:**
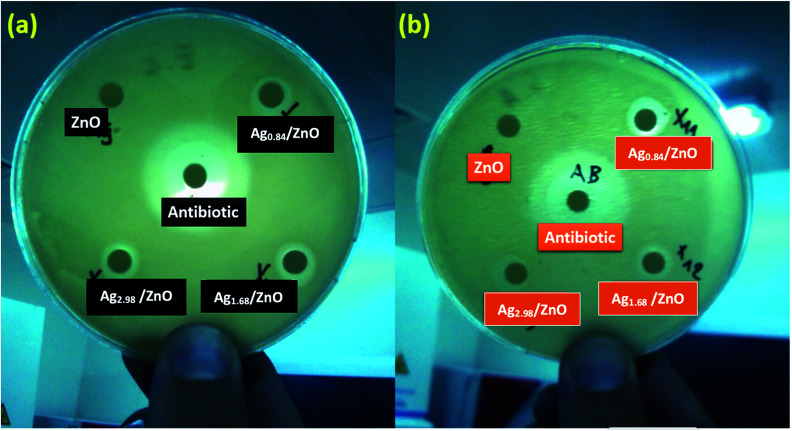
Zones of inhibition for 2000 μg L^−1^ tests for ZnO, Ag_0.84_/ZnO, Ag_1.68_/ZnO, and Ag_2.98_/ZnO towards Gram-negative bacteria. (a) *E. coli* and (b) *S. aureus*.

Aiming to evaluate the influence of ultrasonic energy on the rate constant of the 4-NP reduction to 4-AP, two experiments with and without ultrasonic activation were performed. Fig. 11 presents the kinetic evolution of 4-NP reduction with and without ultrasound agitation. According to Fig. 10, ultrasound can be considered as a very effective intrinsic energy leading to an increase of the homogeneous dispersion of the catalyst in the solution.

ln(*A*_0_/*A*_t_) as a function of time for the two experiments is also plotted in Fig. 12. From the results shown in Fig. 11, we clearly see that ultrasound greatly accelerates the 4-NP reduction reaction from the first minute with a rate constant of 0.47 min^−1^. After a few minutes of reaction, the rate constant is found to decrease to 0.13 min^−1^, and the reaction ends at 4–5 min. In the case of the reaction without ultrasound activation, the rate constant remains constant at 0.05 min^−1^.

### 3.3. Antibacterial activity

Bactericidal activity of metal oxide nanoparticles in part depends on size, stability, and concentration in the growth medium. While growing in medium amended with nanoparticles, the bacterial population growth can be inhibited by specific nanoparticle interactions. From the photographs in Fig. 12a and b, corresponding to the zone of inhibition of *E. coli* and *S. aureus*, respectively, we can clearly observe a slight zone of inhibition (ZOI) formed by all the as-prepared nanohybrid Ag/ZnO materials, and it is larger compared to that formed by pure ZnO, signifying that the Ag/ZnO nanohybrid is an effective antibacterial material for both *E. coli* and *S. aureus*.

**Fig. 13:**
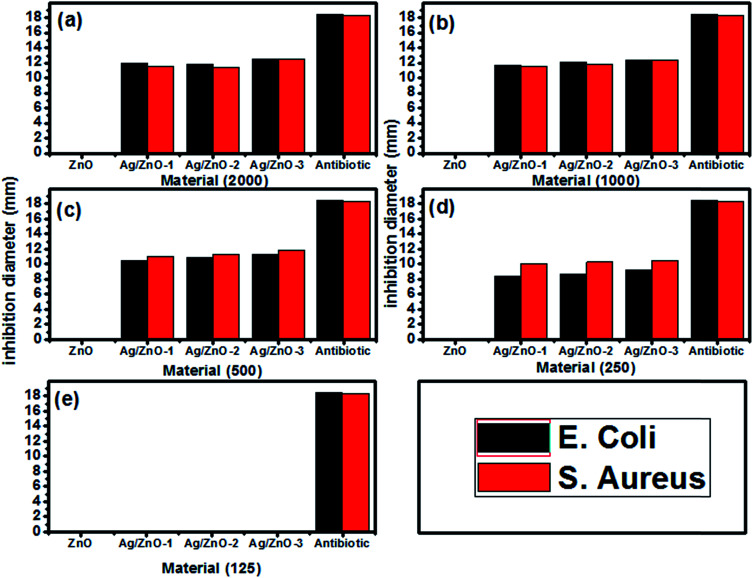
Histograms of the inhibition diameter zone of the as-synthesized materials against *E. coli* and *S. aureus* at different concentrations: (a) 2000, (b) 1000, (c) 500 (d) 250 and (e) 125 μg L^−1^.

The antibacterial activity of the as-prepared materials was tested through the inhibition of *E. coli* and *S. aureus*. Fig. 13 shows that the inhibition of the growth of both bacteria is proportional to the loading of Ag in the Ag/ZnO nanohybrids. The maximum inhibition of this nanoparticle is achieved at a concentration of 500 and 1000 μg ml^−1^ Ag_2.98_/ZnO in *S. aureus* and *E. coli*, respectively. Studies by several groups have shown that the functional activity and consequent toxicity of ZnO nanoparticles may be influenced by particle size and concentration which is inversely proportional to the size and concentration of ZnO nanoparticles used against *S. aureus* and *E. coli*^66^, which showed antibacterial activity of Ag/ZnO on both the strains studied, *E. coli* and *S. aureus*. Another study showed that Ag/ZnO synthesized by a microwave method has antibacterial activity against *S. aureus* when it has no effect on *E. coli*.^67^

## 4. Conclusion

In the present study, Ag/ZnO nanohybrid materials were successfully prepared with different silver loadings *via* an alginate gelation method previously published by our group. The Ag/ZnO nanohybrid materials are stable and well dispersed in aqueous solution. The Ag/ZnO nanohybrid material was successfully characterized by several techniques to confirm the incorporation of the accurate amount of silver with mesoporous morphologies and optical properties. Additionally, our nanohybrid materials were confirmed to be bifunctional nanomaterials thanks to the interaction between the metallic silver and ZnO semiconductor (metal/semiconductor). Indeed, the Ag/ZnO nanohybrid materials showed efficient catalytic and sonocatalytic activity for the conversion of 4-nitrophenol to 4-aminophenol using NaBH_4_ as a reducing agent in aqueous solution.

## Conflicts of interest

There are no conflicts to declare.

## Supplementary Material

NA-001-C9NA00075E-s001
