# Supplementary material for: Highly efficient catalytic/sonocatalytic reduction of 4-nitrophenol and antibacterial activity through a bifunctional Ag/ZnO nanohybrid material prepared via a sodium alginate method
Source: Nanoscale Adv. 2019 Jul 2;1(8):3151–63. doi: 10.1039/c9na00075e (PMC9417202; doi:10.1039/c9na00075e)
Supplement: NA-001-C9NA00075E-s001 [file NA-001-C9NA00075E-s001.pdf]

Electronic Supplementary Material (ESI) for Nanoscale Advances.
This journal is © The Royal Society of Chemistry 2019

Electronic supplementary material

Highly efficient catalytic/sonocatalytic reduction of 4-nitrophenol and antibacterial activities through bifunctional Ag/ZnO nanohybrid material made-up via sodium alginate method

Hicham Abou Oualid[a,b], Othmane Amadine[a], Younes Essamlali[a] , Issam Meftah Kadmiri[c] , Hicham El Arroussi[c], Mohamed Zahouily[a,b,*]

[a.] VARENA Center, MAScIR Foundation, Rabat Design, Rue Mohamed El Jazouli, Madinat El Irfane 10100-Rabat, Morocco.

[b.] Laboratoire de Matériaux, Catalyse et Valorisation des Ressources Naturelles, URAC 24, FST, Université Hassan II-Casablanca BP, 146, 20650, Morocco.

[c.] *Green Biotechnology Center, Moroccan Foundation for Advanced Science, Innovation and Research, Rabat Design Center, Rabat, Morocco*

*Correspondence:m.zahouily@mascir.com ; Tel. +212661416359

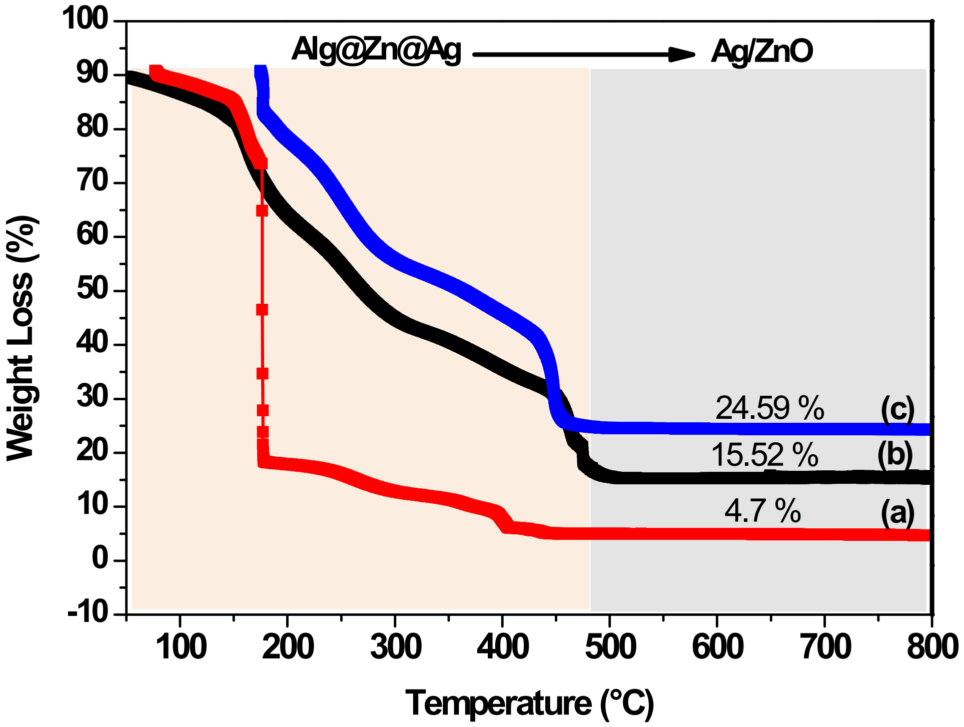

Fig. A1 : TGA of Alg@Zn@Ag1 (a), Alg@Zn@Ag2 (b) and Alg@Zn@Ag3 (c) under air.

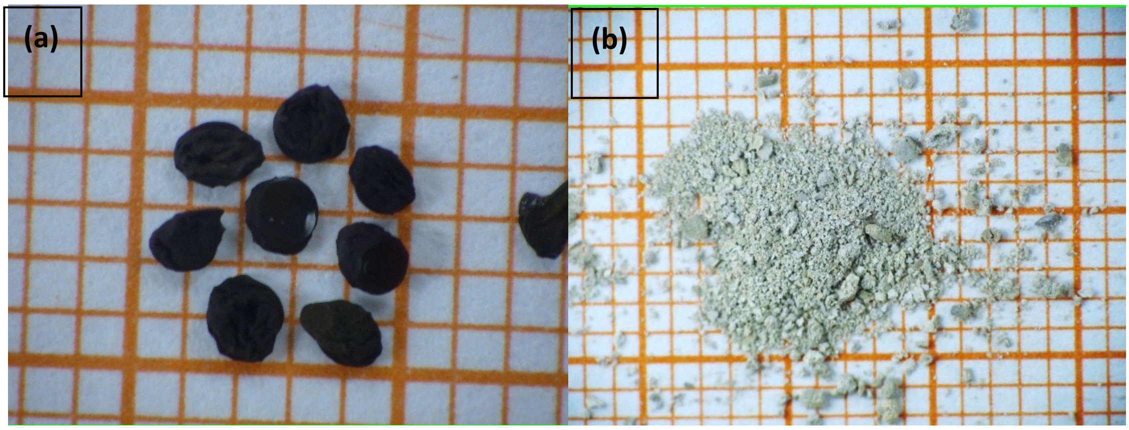

Fig. A2 : Photo of the Alg@Zn@Ag beads(a) and (b) Ag/ZnO powder.

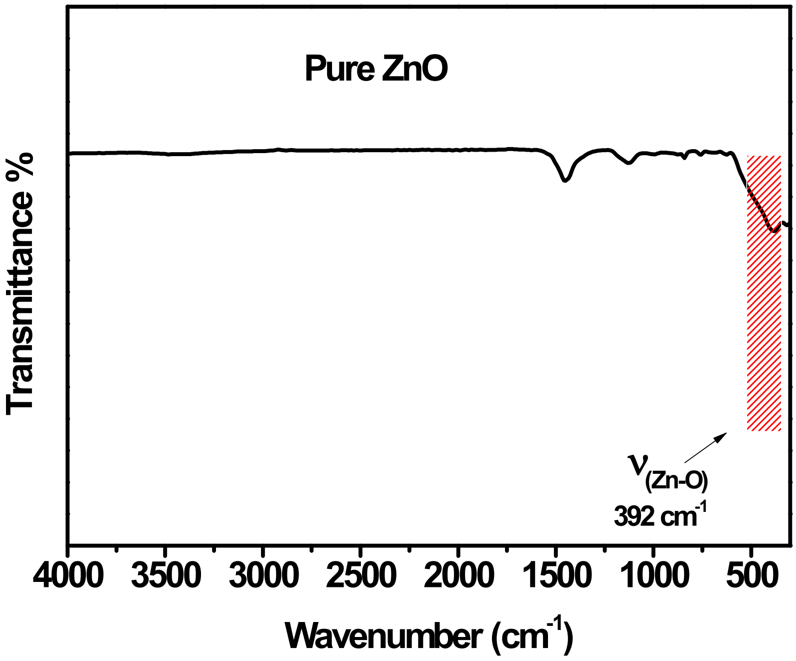

Fig. A3: FTIR analysis of pure zinc oxide fabricated by the same procedure.

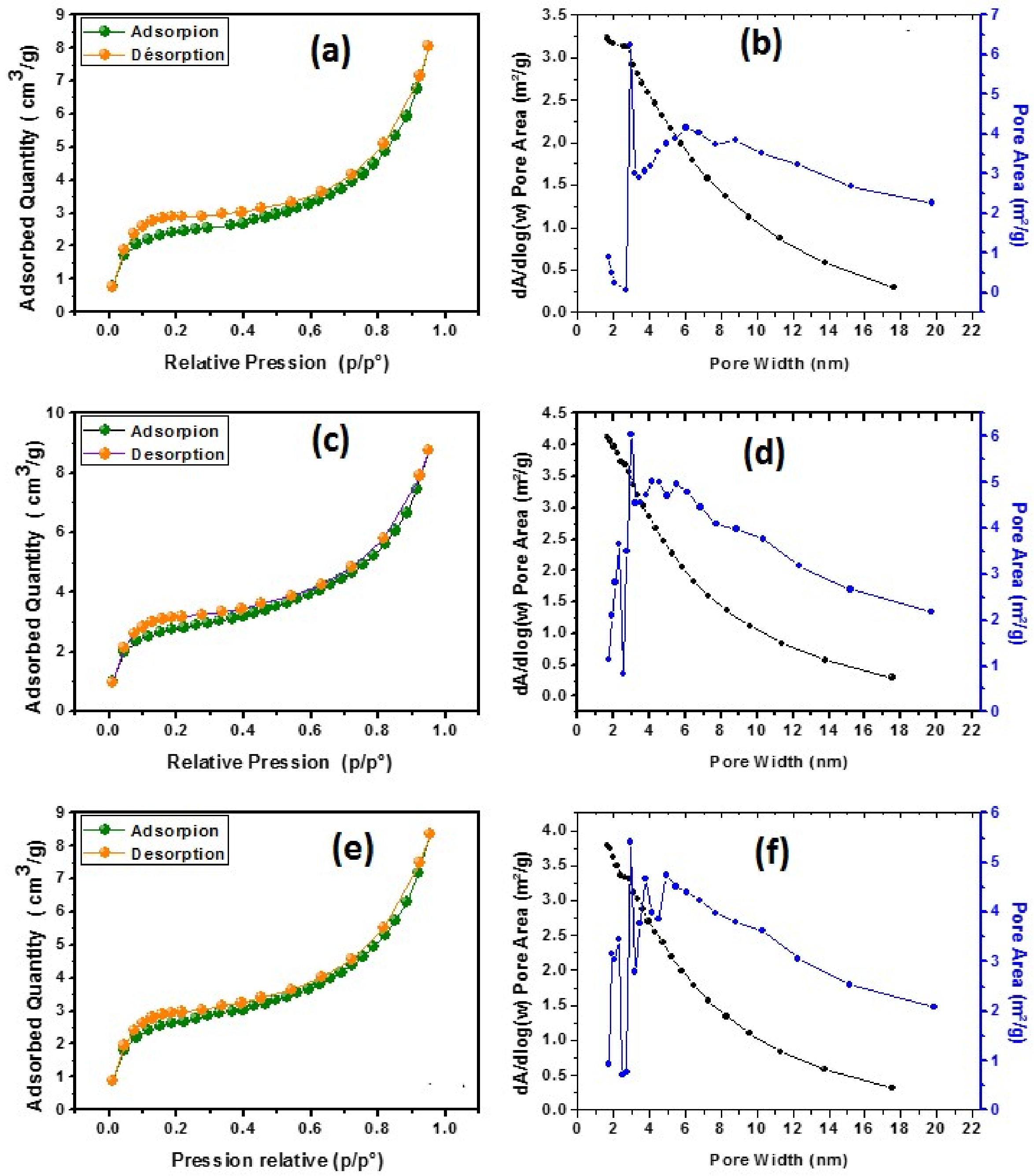

Fig. A4: N_2 adsorption-desorption (isotherms) and BJH pore size distribution of $Ag_{0.84}$/ZnO (a, b), $Ag_{1.68}$/ZnO (c, d) and $Ag_{2.98}$/ZnO (e, f), respectively.

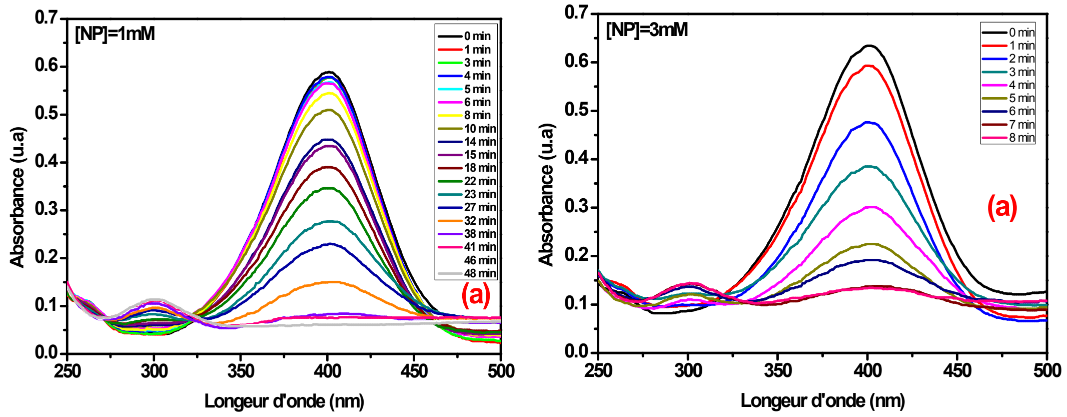

Fig. A5: UV–Vis spectra evolution of solutions of 4-NP in the presence of $NaBH_4$ in different concentration of 4-NP.

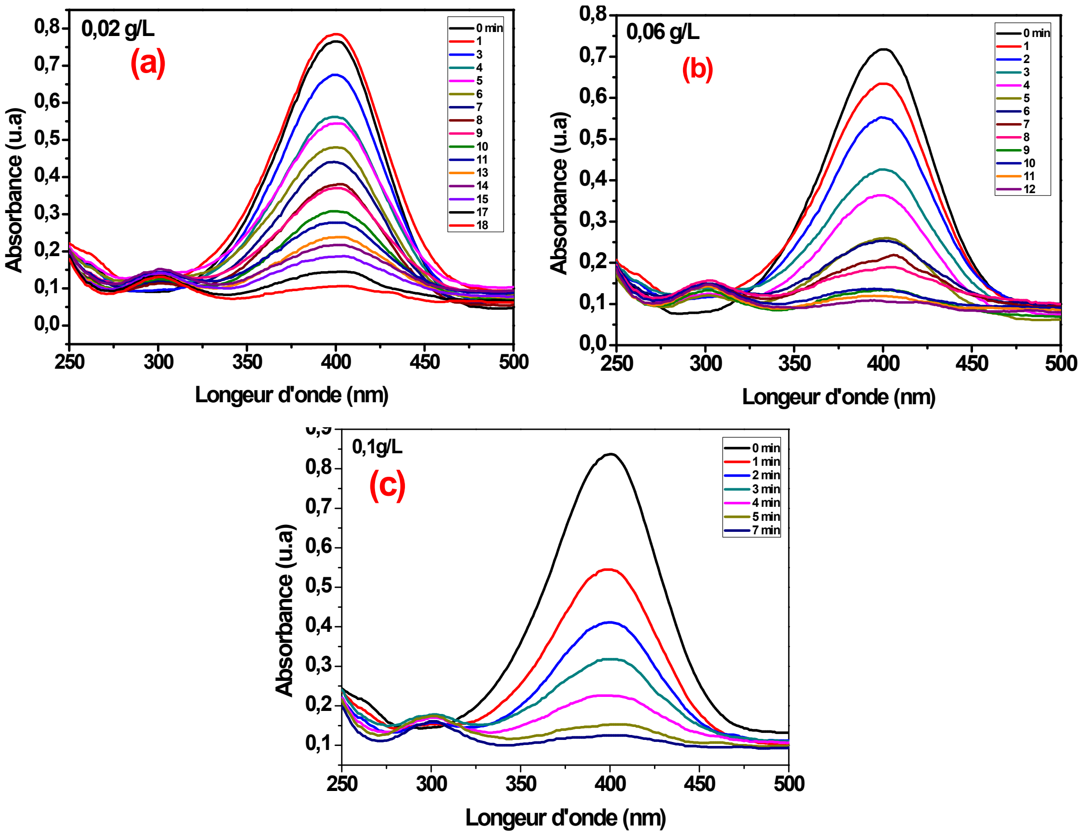

Fig. A6: UV–Vis spectra evolution of solutions of 4-NP in the presence of $NaBH_4$ in different mass concentrations of Ag/ZnO catalyst.

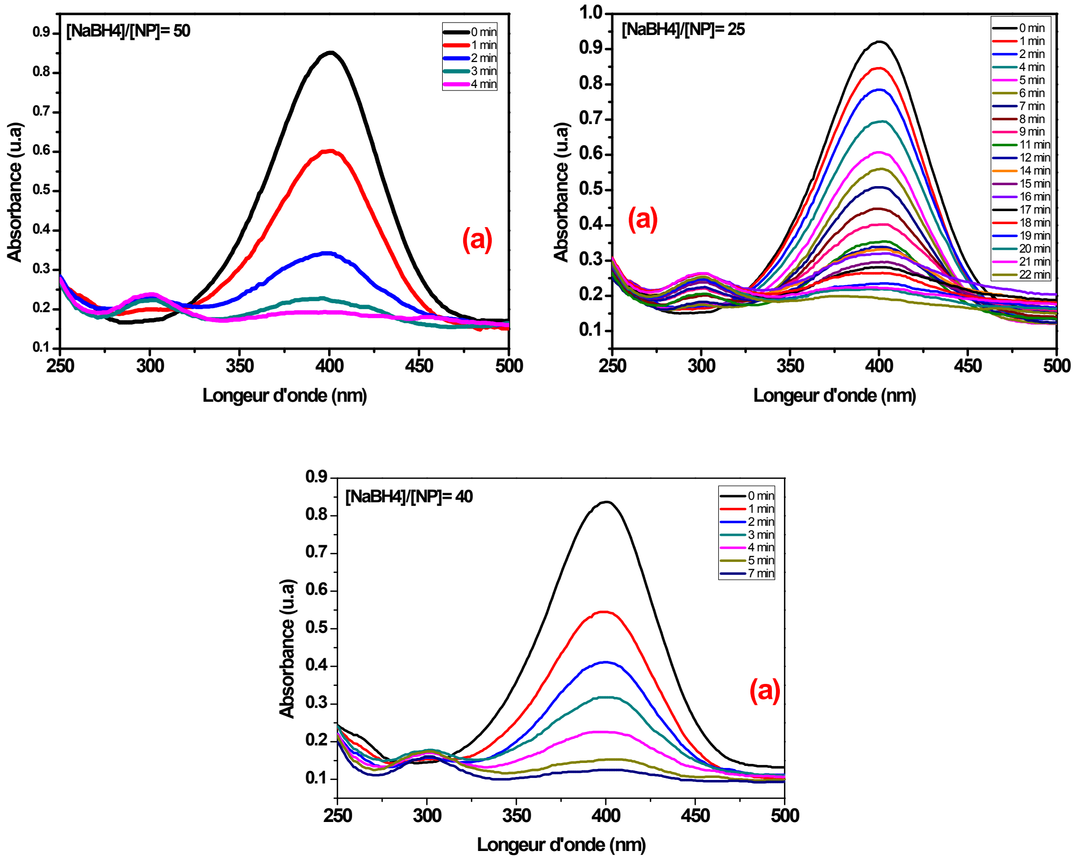

Fig. A7: UV–Vis spectra evolution of solutions of 4-NP in the presence of $NaBH_4$ in different in different R ratio.
